# Deterministic nanoantenna array design for stable plasmon-enhanced harmonic generation

**DOI:** 10.1515/nanoph-2022-0365

**Published:** 2022-10-24

**Authors:** Tae-In Jeong, Dong Kyo Oh, San Kim, Jongkyoon Park, Yeseul Kim, Jungho Mun, Kyujung Kim, Soo Hoon Chew, Junsuk Rho, Seungchul Kim

**Affiliations:** Department of Cogno-Mechatronics Engineering, College of Nanoscience and Nanotechnology, Pusan National University, Busan 46241, Republic of Korea; Department of Mechanical Engineering, Pohang University of Science and Technology (POSTECH), Pohang 37673, Republic of Korea; Department of Optics and Mechatronics Engineering, College of Nanoscience and Nanotechnology, Pusan National University, Busan 46241, Republic of Korea; Department of Chemical Engineering, Pohang University of Science and Technology (POSTECH), Pohang 37673, Republic of Korea; POSCO-POSTECH-RIST Convergence Research Center for Flat Optics and Metaphotonics, Pohang 37673, Republic of Korea; National Institute of Nanomaterials Technology (NINT), Pohang 37673, Republic of Korea

**Keywords:** nanophotonics, nonlinear optics, plasmonic field enhancement, third-harmonic generation

## Abstract

Plasmonic nanoantennas have been extensively explored to boost nonlinear optical processes due to their capabilities to confine optical fields on the nanoscale. In harmonic generation, nanoantenna array architectures are often employed to increase the number of emitters in order to efficiently enhance the harmonic emission. A small laser focus spot on the nanoantenna array maximizes the harmonic yield since it scales nonlinearly with the incident laser intensity. However, the nonlinear yield of the nanoantennas lying at the boundary of a focused beam may exhibit significant deviations in comparison to those at the center of the beam due to the Gaussian intensity distribution of the beam. This spatial beam inhomogeneity can cause power instability of the emitted harmonics when the lateral beam position is not stable which we observed in plasmon-enhanced third-harmonic generation (THG). Hence, we propose a method for deterministically designing the density of a nanoantenna array to decrease the instability of the beam position-dependent THG yield. This method is based on reducing the ratio between the number of ambiguous nanoantennas located at the beam boundary and the total number of nanoantennas within the beam diameter to increase the plasmon-enhanced THG stability, which we term as the ratio of ambiguity (*ROA*). We find that the coefficient of variation of the measured plasmonic THG yield enhancement decreases with the *ROA*. Thus, our method is beneficial for designing reliable sensors or nonlinear optical devices consisting of nanoantenna arrays for enhancing output signals.

## 1 Introduction

The strength of an excitation field is the key factor in performing nonlinear experiments at a detectable level owing to its power-law scaling, which is proportional to the *n*th power of the field intensity for *n*th order nonlinear phenomena [1, 2]. Surface plasmons (SPs) enable such a requirement to be met by generating strongly enhanced local electromagnetic fields around the surfaces of metallic nanoantennas [3–8]. In previous experiments involving SPs, two-dimensional or three-dimensional nanoantenna array configurations were preferred over single, isolated nanoparticles since the size of a nanostructure is on the subwavelength scale, which is much smaller than the size of a focused incident beam, thus allowing a collective plasmonic excitation within the large number of nanoantennas [9–13]. Furthermore, the focused beam was adjusted to be oriented precisely along the optical axis to maximize the incident field strength at the nanoantennas [14, 15]. However, the power instability of harmonic generation due to a shift of the lateral beam position in a nanostructure-array configuration has not yet been investigated, which could be caused by subtly different laser illumination conditions. Typically, the focused beam has a lateral Gaussian intensity distribution, implying that the field strength decreases steeply from the center to the boundary of the beam. This abrupt decrease can cause considerably fluctuating harmonic yields between the nanoantennas located at the center and those at the boundary of the beam. Moreover, microscale vibrations or mechanical drifts under ambient conditions, which can cause a mechanical instability in harmonic generation, are inevitable. Therefore, a robust design that is immune to such mechanical drift and noise is desirable for the practical application of nonlinear nanophotonic devices.

**Figure 1:**
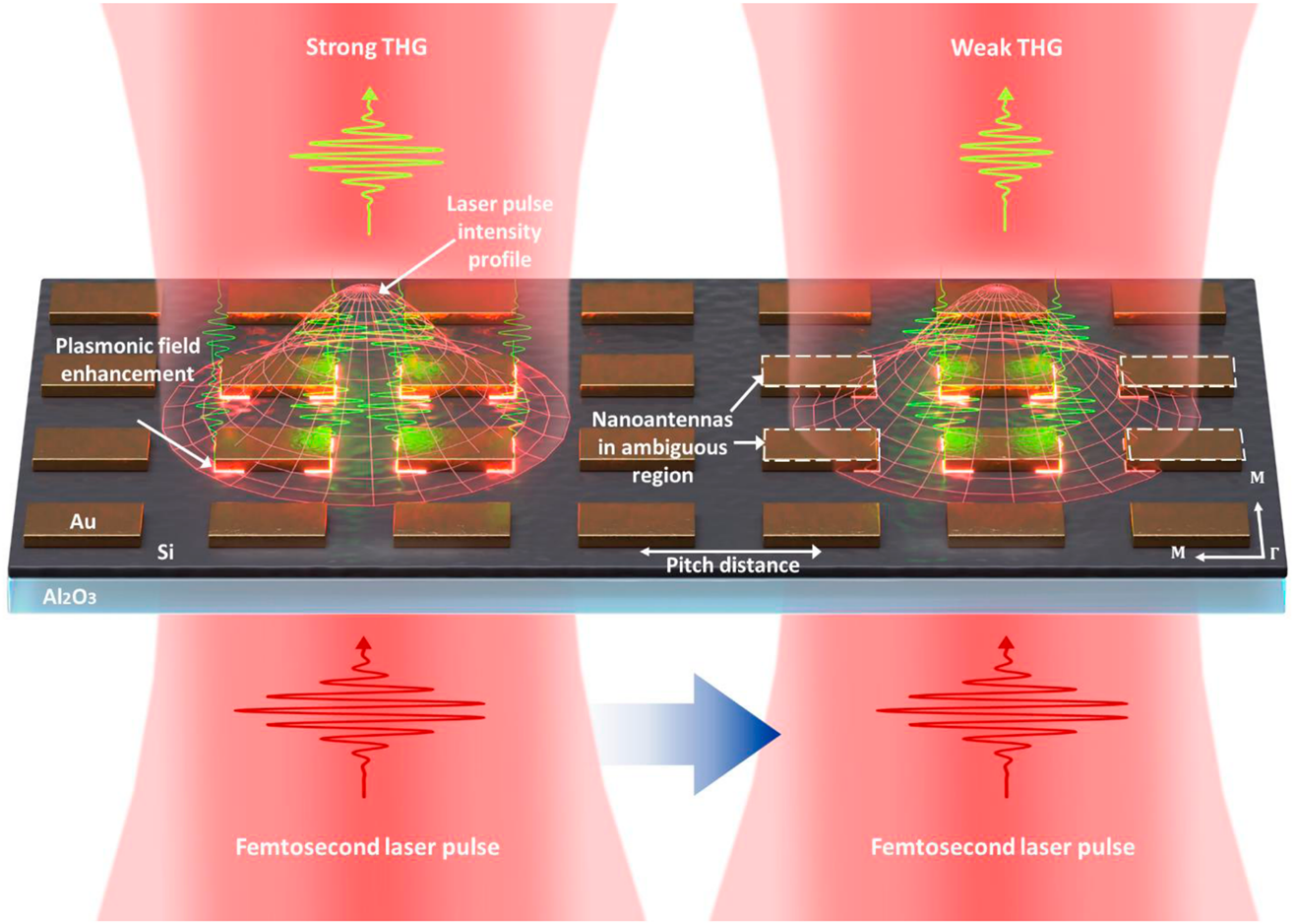
Illustration of the power instability of plasmon-enhanced THG in a nanoantenna array. Because the intensity of a focused beam is not spatially homogenous, adjacent nanoantennas are subjected to different laser field strengths when the laser beam is shifted from the left to right side, causing the third-harmonic yield to vary.

Figure 1 schematically illustrates the third-harmonic generation (THG) from a Si film enhanced by an Au nanoantenna array upon femtosecond laser illumination at two different focused laser beam positions. The plasmon-enhanced THG yield is stronger when the nanoantennas are located within the focused beam area (left) and becomes weaker when the beam is shifted to an ambiguous region where some parts of the nanoantennas are outside of the beam area (right) even though the fabricated shapes of all the nanoantennas are identical. This shows that the THG yield can vary when the beam shifts with respect to the nanoantenna array since they are exposed to different laser field strengths as given by the Gaussian beam profile. In this study, we experimentally demonstrate that a lateral shift of the focused beam with respect to the nanoantenna array is responsible for the instability observed in the plasmon-enhanced THG yield. Furthermore, this effect becomes more obvious as the pitch distance of the array increases to the scale of the focused beam size because adjacent nanoantennas are subjected to greater changes of the input field strength. In that case, the nanoantennas at the boundary of the focused beam are not fully operated as plasmon-enhanced THG emitters and therefore significantly affect the reliability of the total THG yield.

## 2 Experimental setup and nanoantenna array design

**Figure 2:**
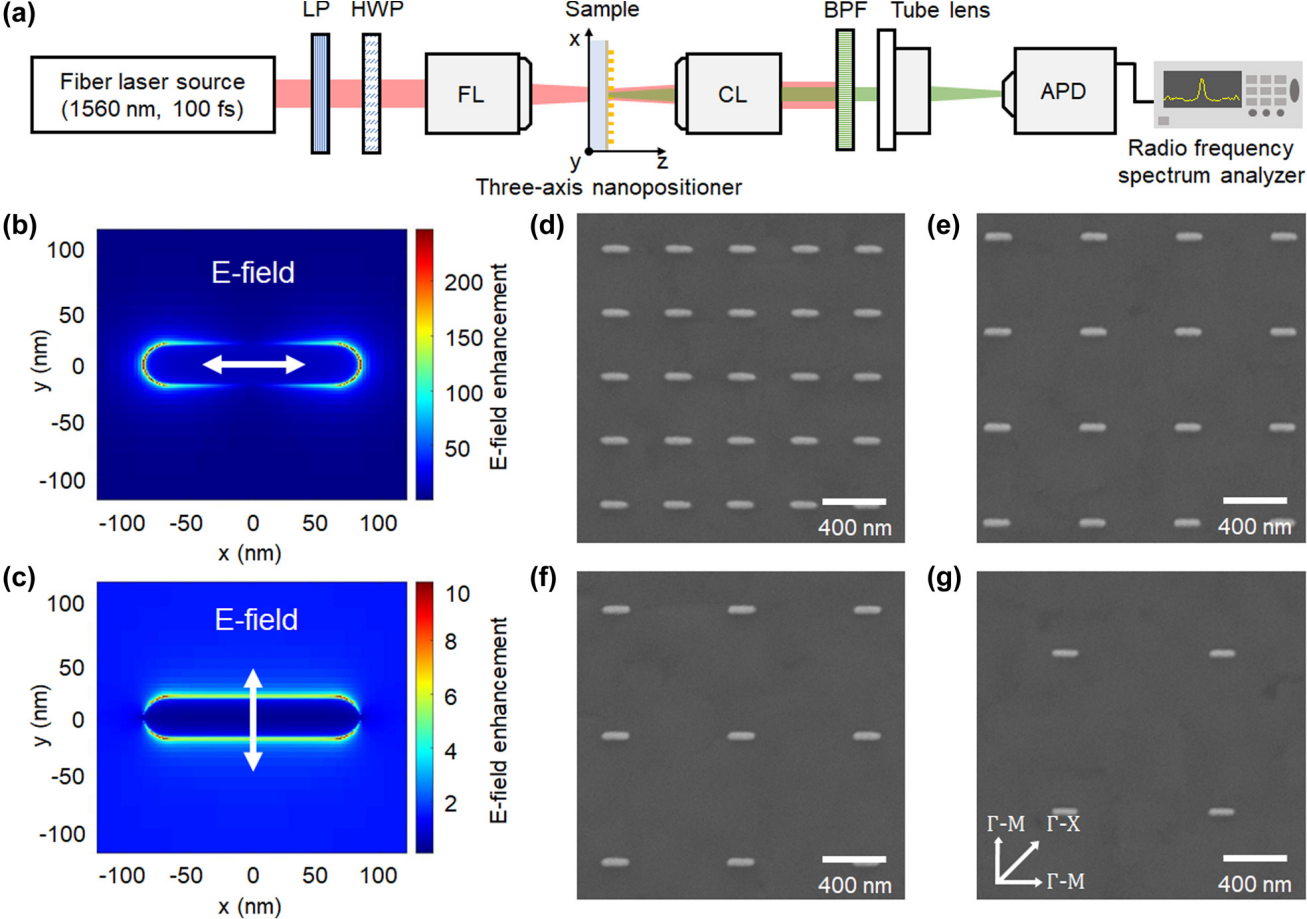
Experimental setup and Au nanoantenna array designs. (a) Schematic diagram of the plasmon-enhanced THG setup for measuring the third-harmonic signal based on lateral position shift. LP, linear polarizer; HWP, half-wave plate; FL, focusing lens; CL, collecting lens; BPF, bandpass filter; APD, avalanche photodetector. FDTD simulation results showing time-averaged plasmonic E-field enhancement for laser polarization (b) parallel and (c) perpendicular to the major axis of a nanoantenna, respectively. Scanning electron microscope images of Au nanoantenna arrays fabricated on a single-crystalline Si film deposited on a sapphire substrate, with pitch distances of (d) 400, (e) 600, (f) 800, and (g) 1000 nm. The reciprocal lattice points of the Si film are marked in (g).

An erbium-doped fiber laser source (C-Fiber High Power, Menlo Systems) emitting 100 fs infrared laser pulses with a central wavelength of 1560 nm and at a repetition rate of 100 MHz was used for the plasmon-enhanced THG experiments, as shown in Figure 2a. The femtosecond pulses were focused onto an Au nanoantenna array sample to reach sub-terawatt-level peak intensities (<0.1 TW cm^−2^) below the laser damage threshold. In order to compare the effect of beam size on the same nanoantenna array, two objective lenses with different numerical apertures (NAs) of 0.25 and 0.4 were used to focus the laser beam down to radii of 3.15 and 2.35 μm, respectively. A combination of a half-wave plate and a linear polarizer was used to rotate the incident laser polarization and also vary the laser power for polarization- and intensity-dependent THG yield measurements. The sample was mounted on a three-axis nanopositioner equipped with an imaging system for precise alignment between the laser focal spot and the nanoantenna array. The sample consisted of Au nanoantenna arrays fabricated on a 500 nm-thick single-crystalline Si film deposited on a 460 μm-thick R-plane Al_2_O_3_ substrate. Such thin Si film was chosen to minimize the THG contribution from the bulk of the Si film while optimizing the plasmon-enhanced THG from the interface between the film and the nanoantennas. The third harmonic emitted from the sample together with the fundamental laser was collected in transmission through the substrate with an objective lens (NA = 0.4). A bandpass filter (FGS900-A, Thorlabs) was used behind the collecting lens to block the fundamental laser but transmit the third harmonic. The transmitted third-harmonic signal was then detected by an avalanche photodetector (APD 210, Menlo Systems) in combination with a radio frequency spectrum analyzer (DSA815, RIGOL) at the repetition frequency of the laser to eliminate environmental noises at other frequencies.

The dimensions of the Au nanoantenna were numerically determined to enable strong absorption at the central wavelength of the femtosecond pulses, resulting in strong plasmonic field enhancement when the laser polarization was parallel to the major axis of the nanoantenna (see Supporting Information Section 1) [16–18]. The designated nanoantenna design had a length of 165 nm, width of 35 nm, and thickness of 40 nm with rounded corners, considering the fabrication limits of e-beam nanolithography. Figure 2b and c show the local field distributions of the designated nanoantenna at the interface between the nanoantenna and Si film calculated by the finite-difference time-domain (FDTD) method (Lumerical Solutions) with orthogonal laser polarizations. A maximum field enhancement exceeding 200 at the rounded corners of the nanoantenna is obtained when the laser polarization is aligned parallel to the major axis of the nanoantenna. In contrast, the enhancement is less than 10 when the polarization is perpendicular to the major axis of the nanoantenna; however, this is still sufficient for observing the plasmon-enhanced THG. In total, nanoantenna arrays with four different pitch distances of 400, 600, 800, and 1000 nm were fabricated on the same substrate using e-beam lithography to minimize the experimental errors caused by variations in fabrication parameters such as Au film thickness or Si substrate quality (see Supporting Information Section 2 for the detailed fabrication process and Section 3 for larger fields of view of the nanoantenna arrays), where the nanoantenna length (major axis) was parallel to the [110] orientation of the Si lattice (the Γ–M direction in the Brillouin zone), as shown in Figure 2d–g. The entire fabricated nanoantenna array size of each pitch distance was set to 600 μm × 600 μm, which was approximately 100 times larger than the laser focal spot, in order to exclude any effects from the boundary of the fabricated area. The sample fabrication was monitored using scanning electron microscopy on additional reference nanoantenna arrays to confirm the uniformity of the nanoantenna dimensions used for experiments.

## 3 Results and discussion

### 3.1 Characterization of plasmon-enhanced THG

**Figure 3:**
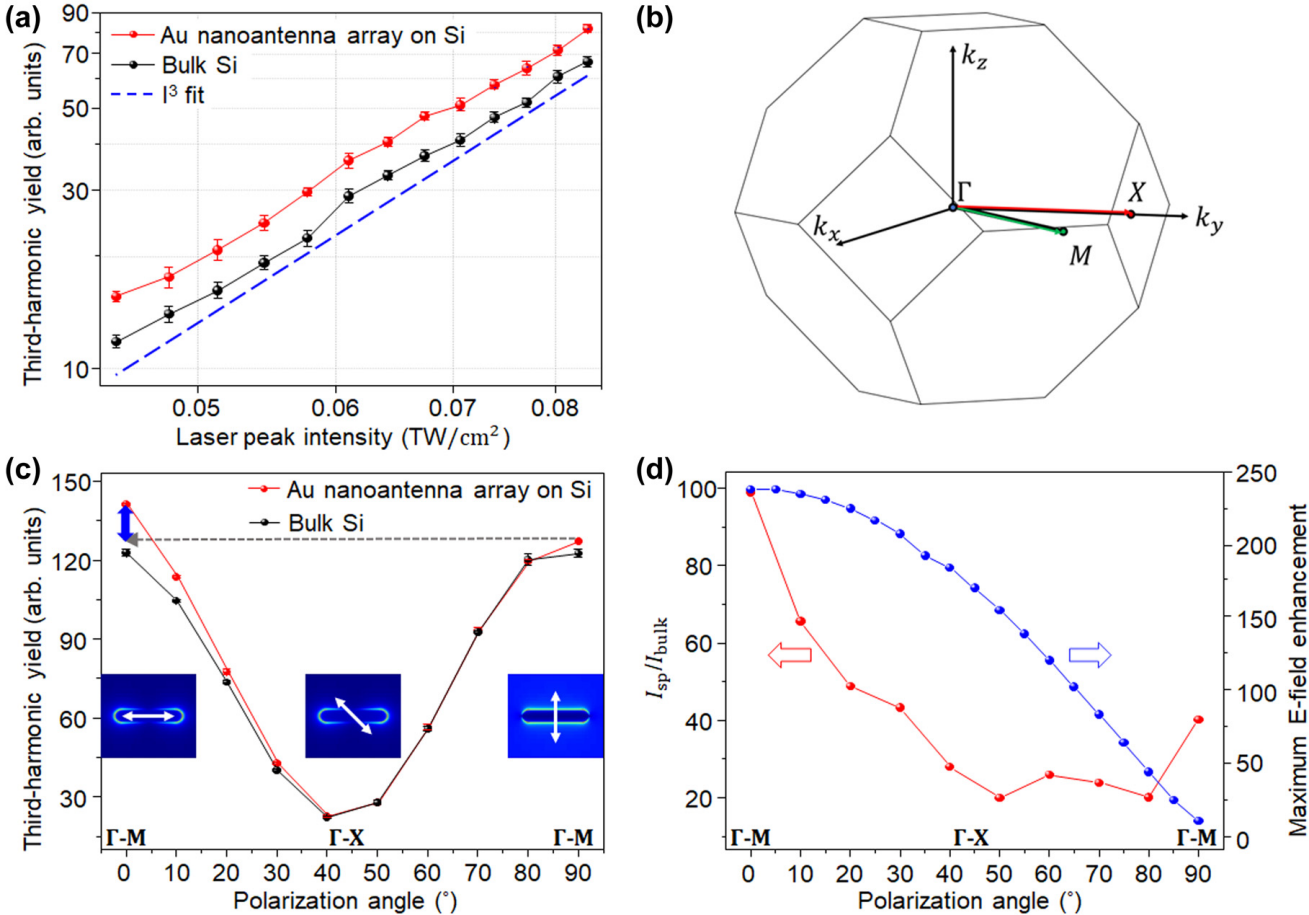
Laser intensity and polarization dependences of plasmon-enhanced THG for a 400 nm-pitch Au nanoantenna array on a Si film and unpatterned bulk Si. (a) Third-harmonic yield as a function of laser intensity on a log–log scale for the nanoantenna array on Si and for bulk Si without any nanoantennas with the laser polarization parallel to the Γ–M direction. The harmonic yield at every pump laser intensity was measured for five times with a fixed laser focal spot position. The dashed blue line represents an *I*^3^ fit where *I* is the laser intensity. (b) First Brillouin zone of the FCC lattice of Si. (c) Modulation of the third-harmonic yield as the laser polarization is rotated, with maximum emission when the polarization is parallel to the Γ–M directions in the bulk Si. The white arrows in the three FDTD simulation images represent different laser polarizations with respect to a nanoantenna. The blue arrow shows the difference in plasmon-enhanced harmonic yield between polarization angles of 0° and 90°. (d) Plasmonic THG yield enhancement, *I*_sp_/*I*_bulk_ (red curve), and FDTD-simulated maximum plasmonic field enhancement (blue curve) as functions of the polarization angle.

In Figure 3, we show the comparison of intensity and polarization dependences of the plasmon-enhanced THG for the Au nanoantenna array with a pitch distance of 400 nm and unpatterned bulk Si. Only the nanoantenna array of 400 nm-pitch-distance was used to characterize the THG since it gave the highest third-harmonic yield compared to other pitch distances. Si is a face-centered cubic (FCC) material that strongly generates odd-order harmonics at the air-Si interface [19]. The THG yield follows an *I*^3^ power law with increasing pump laser intensity, *I*, for both the 400 nm-pitch nanoantenna array and unpatterned bulk Si, when the laser polarization was aligned along the major axis of the nanoantennas (see Figure 3a). The laser focal spot was optimized to maximize the third-harmonic yield. Notably, the THG yield emitted from the nanoantenna array was ∼15% stronger than the emission from the unpatterned bulk Si due to field enhancement, even though the nanoantennas covered only ∼3% of the total area. Figure 3b depicts the first Brillouin zone of the FCC lattice of the Si substrate with four-fold symmetry (Γ–M and Γ–X directions). The third-harmonic yield in the bulk Si without nanoantennas was modulated with four-fold symmetry (a periodicity of 90°), which is the same as the periodicity of the crystal orientation in Si (black curve), as shown in Figure 3c. The third-harmonic yield was maximized when the laser polarization was oriented along the Γ–M direction of the Brillouin zone, but the yield was suppressed continuously when the laser polarization was rotated from the Γ–M to the Γ–X direction. Interestingly, a similar tendency but with a distinct deviation in the harmonic yield was seen in two Γ–M directions in the nanoantenna array (red curve); the harmonic yield at a polarization angle of 0° (parallel to the major axis of the nanoantenna) was stronger than that at a polarization angle of 90° (perpendicular to the major axis of the nanoantenna) for the same Γ–M direction [9, 20]. This is because the strongest field enhancement evidently occurs at a polarization angle of 0°, which is consistent with the simulation results (see Figure 2b and c).

This experimental data shows less polarization dependence in the field enhancement factor, while the polarization effect is rather distinct in the simulation. Physically, THG is induced inside the Si layer, not at the Au nanoantenna surface, which indicates that the plasmonic field enhancement at the Si surface may not effectively amplify the THG yield. We further simulated the plasmonic field distribution at a depth of 5 nm inside the Si surface (see Supporting Information Section 4). Interestingly, the enhanced field when the laser polarization is parallel to the major axis of the nanoantenna is distributed around the boundary of and underneath the Au nanoantenna, which could infer that the some of generated THG signal would be blocked by the Au nanoantenna. On the other hand, the enhancement field for the laser polarization perpendicular to the major axis of a nanoantenna is distributed outside of the Au nanoantenna, and the area of the enhancement field is slightly larger than that of the parallel polarization state. These effects, including imperfect numerical modeling of the fabricated nanoantenna, would result in lower THG yield ratio and less polarization dependence for the two polarization states.

### 3.2 Estimate of plasmonic THG yield enhancement

The plasmon contribution to the harmonic yield was further analyzed by calculating the harmonic yield per unit area in the bulk Si and nanoantenna array regions. The formula for extracting the third-harmonic power per unit area contributed by the bulk Si is

(1) $$I_{\text{bulk}}=\frac{P_{\text{bulk}}}{\pi \times r^{2}},$$

where *P*_bulk_ is the measured third-harmonic power of the bulk Si without a nanoantenna array and *r* is the radius of the focused beam. Here, when each nanoantenna is assumed to emit third-harmonic radiation of equal intensity, the plasmon-enhanced third-harmonic power per unit area can be extracted as follows:

(2) $$I_{\text{sp}}=\frac{P_{\text{sp}}-\left(I_{\text{bulk}}\times A_{\text{inactive}}\right)}{A_{\text{active}}\times N_{\text{total}}},$$

where *P*_sp_ is the measured third-harmonic power of the nanoantenna array, and *A*_inactive_ and *A*_active_ denote the surface areas contributing to the bulk and plasmon-enhanced THG from the nanoantenna array, respectively (see Supporting Information Section 5 for the detailed calculations). The total number of nanoantennas located in the focused beam, *N*_total_, was assumed to be

(3) $$N_{\text{total}}=\frac{\pi \times r^{2}}{d^{2}},$$

where *d* denotes the pitch distance of the fabricated nanoantenna array. Using Eqs. (1)–(3) above, the plasmon-enhanced THG yield enhancement, *I*_sp_/*I*_bulk_, which is the ratio between the plasmon-enhanced and bulk THG power per unit area, can be plotted as a function of laser polarization, as shown in Figure 3d. Note that we assume each nanoantenna within the focused beam area generates an equivalent third-harmonic emission here, which is common for evaluating the plasmonic field enhancement in nonlinear experiments [9, 15]. The THG yield enhancement has a maximum of ∼100 at a laser polarization angle of 0° and decreases to ∼25 when the polarization angle approaches 45°, then increases again up to ∼40 with some fluctuations in between for polarization angles of 45°–90° (red curve). On the other hand, the simulated maximum plasmonic enhancement decreases continuously as the polarization angle changes from 0° to 90° (blue curve). One would expect a continuously decreasing THG yield enhancement for polarization angles of 45°–90° due to decreasing plasmonic enhancement from 45° to 90°; however, we observe an increase in the THG yield enhancement above 45° (red curve in Figure 3d) due to the effective area change of plasmonic field enhancement with respect to the polarization (black curve in Figure 3c). In other words, the combination of both the reduced maximum plasmonic field enhancement and increased effective plasmonic THG area contribute to the plasmon-enhanced THG yield.

### 3.3 Instability of plasmon-enhanced THG

**Figure 4:**
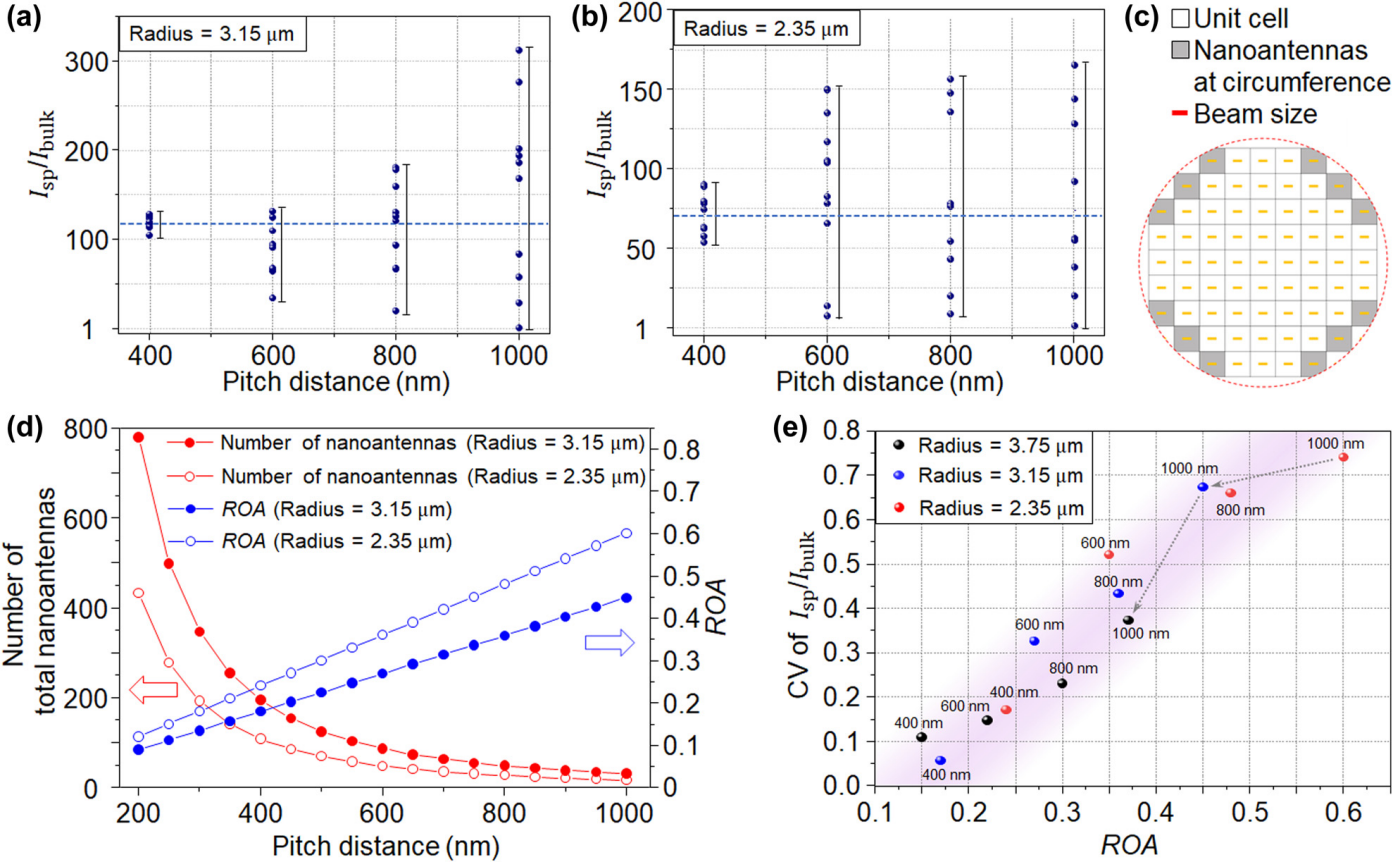
Plasmon-enhanced THG instability and evaluation of the *ROA*. Plasmonic THG yield enhancement measured at 10 different positions for each pitch distance with (a) radius = 3.15 μm and (b) radius = 2.35 μm. The dashed blue line represents the mean value of the THG yield enhancement for a nanoantenna array with a pitch distance of 400 nm. (c) Schematic illustration of the analytical die count estimation method. (d) Numerically calculated total number of nanoantennas excited by the laser beam (red curves) and the *ROA* values calculated based on the analytical die count estimation method (blue curves) for varying pitch distances with radius = 3.15 μm and radius = 2.35 μm. (e) CV of the THG yield enhancement as a function of the *ROA* with radius = 3.75, radius = 3.15 and radius = 2.35 μm. Note that radius = 3.75 μm (NA = 0.25*) resulted in a larger focused beam radius since a smaller laser beam diameter at input aperture was used with the same objective lens of radii = 3.15 μm (NA = 0.25). The dotted grey arrows are a guide to the eye, showing the *ROA* decreases with increasing focused beam radius for a pitch distance of 1000 nm, for instance.

We observed fluctuations in the extracted plasmonic THG yield enhancement from all nanoantenna arrays (pitch distances of 400, 600, 800, and 1000 nm) upon laser illumination even with fixed laser focus conditions. Therefore, we systematically investigated the cause of the instability found in the THG yield by using two different focused laser beam radii on the sample (Figure 4a and b). Focused beam radii of 3.15 and 2.35 μm were obtained using objective lenses with different NA = 0.25 (Figure 4a) and NA = 0.4 (Figure 4b), respectively. The laser polarization was aligned parallel to the major axis of the nanoantenna arrays to achieve maximum plasmon-enhanced THG signals. We found that when the sample was constantly shifted in the horizontal direction with respect to the focused beam with an incremental step size of 1/10 of each pitch distance for 10 times, the THG yield enhancement at each position was different, providing a clear evidence that plasmon-enhanced harmonic generation can vary according to the focused beam position on a nanoantenna array. In these experiments, unpredictable mechanical vibrations can cause third-harmonic power fluctuations at each focused beam position. These vibrations can simultaneously induce a fluctuation of the laser beam position along the optical axis, which is not related to the pitch distance of the nanoantenna array. In our experiments, the experimental setup was built on a vibration isolation table and inside an enclosure to minimize external vibrational noises; we verified that the fluctuation of third-harmonic yield by mechanical vibration was negligible for a fixed laser beam position. In addition, the third-harmonic signal was averaged over 1 s for each laser beam position to minimize high-frequency noises; the resultant third-harmonic yield was obtained by accumulating 10^8^ successive pulses. Furthermore, the power instability of our femtosecond laser system did not significantly affect the plasmon-enhanced THG instability over time because the measured power instability of the laser was less than 0.17% over 1 h (Figure S4). If the mechanical vibration or laser stability affects the instability of the third-harmonic generation, this effect should also similarly influence all the experimental cases, such as all the pitch distances. Nevertheless, different increasing third-harmonic power instability was identified for pitch distances of 400, 600, 800, and 1000 nm.

The experimentally observed third-harmonic yield enhancement typically reflects the fabrication quality of nanoantennas regarding to a specified design. However, our results show that it is unclear whether the observed large variation of the extracted THG yield enhancement at different beam positions on the sample is caused by imperfections of the fabrication process or optical misalignment. For radius of 3.15 μm, as the pitch distance increases to 1000 nm, the THG yield enhancement exhibits a strong variation between 1 and 313, which is hard to quantify the plasmonic field enhancement of a nanoantenna. Such a large variation could be detrimental to the reliability and performance of plasmonic nanoantenna arrays. Experimentally, the total *A*_active_ and *A*_inactive_ areas can vary with the lateral beam position shift, which should be applied in calculating *I*_sp_ and *I*_bulk_. Nevertheless, our analysis considered them as the fixed values for the same pitch distance and focused beam size. Because the precise measurement of the laser beam position on the nanoantenna array is complicated during the THG experiment, applying the different active areas for each THG data is practically not easy. In addition, the change of total active area is relatively less than the size of *A*_inactive_ (bulk surface area in nanoantenna array), which does not severely affect the third-harmonic power calculation using Eqs. (1)–(3), while this small active area change causes the total plasmon-enhanced THG yield instability. To investigate this, we calculated the nanoantenna array’s maximum and minimum active areas, (*A*_active_ × *N*_total_) at a focused beam radius of 2.35 μm for each pitch distance (see Supporting Information Section 7). The *A*_active_ for each nanoantenna was acquired from the FDTD simulation, and the total active areas, *A*_active_ × *N*_total,_ were calculated using a graphical software, considering the area of a nanoantenna array where the focused laser beam overlaps. Figure S5 shows the calculated plasmonic THG yield enhancement for the maximum and minimum active areas at each pitch distance. The graph shows that the change in the total active area does not significantly affect the original analysis, as shown in Figure 4b; applying the exact active area for each measurement data does not significantly reduce the third-harmonic power instability in Figure 4a and b.

The method of counting the number of nanoantennas when extracting the THG yield enhancement using Eqs. (1)–(3) is only valid under particular conditions, such as when the pitch distance is sufficiently shorter than the focused beam diameter. When the focused beam size is reduced using a lens of higher NA, the THG yield enhancement instability starts to appear at a shorter pitch distance of 400 nm (Figure 4b), indicating that the pitch distance is closely correlated with the focused laser beam size in determining the onset of the instability. Interestingly, the mean values of the THG yield enhancement are very similar for all pitch distances, and tend to approach the mean value for the shortest pitch distance of 400 nm. Ideally, the measured THG yield enhancement should be the same for all pitch distances since the nanoantennas are not coupled with each other due to their far instance from each other. Moreover, the plasmonic THG yield enhancement is mainly determined by the geometry of a nanoantenna and the complex permittivities of the materials and environment, therefore the change of beam size or pitch distance should not play a role in the THG yield enhancement. When the pitch distance is short enough to minimize the instability effect, the THG yield enhancement should be the same for both NA values. However, since the third-order nonlinear process is highly sensitive to the peak intensity changes of the input laser, e.g., due to pulse broadening caused by dispersion in a thicker lens (higher NA), the THG yield enhancement was reduced for radius of 2.35 μm.

Surface lattice resonances, which constitute the diffractively coupled plasmon resonance modes in a nanoantenna array [21, 22], were not considered in the experimental analysis, although these could affect the efficiency of plasmon-enhanced THG. Surface lattice resonances require the incident light illumination to be spatially coherent over a large area to enable constructive interference of scattered light produced by all the nanoantennas that make up the array. Therefore, a focusing lens with a small NA (e.g., 0.1) is preferred for irradiation to achieve a uniform phase on the array. However, in our experiments, we used focusing lenses with relatively high NAs (0.25 and 0.4), hence did not satisfy this condition to excite the surface lattice resonance modes efficiently [23]. In fact, plasmon-enhanced THG is commonly known to originate from the Si substrate but strongly enlarged by the strong plasmonic fields generated at the interface between the nanoantennas and the substrate. On the other hand, we also considered the possibility of THG yield contributed by the third-order nonlinear responses of Au nanoantennas induced by interband transitions from the 5d band to the 6sp band in Au. It has been reported that the THG yield can be significantly increased by utilizing the third-order nonlinearity of Au which is strongly influenced by its interband transitions in Au nanoantennas fabricated on a glass substrate [24]. However, in our case, we believe that the THG contribution from third-order nonlinearity of Au nanoantennas is negligible since the third-order nonlinear susceptibility of Au is only approximately 1/10 that of Si, hence the THG power of Au is approximately 1/100 or even smaller compared to Si, since it scales with the nonlinear susceptibility squared [25, 26]. In addition, the fabricated Au nanoantennas are of polycrystalline nature, which should result in a lower THG efficiency than for single-crystalline materials.

Therefore, the instability of the THG yield enhancement could only have been induced by the spatial alignment between the focused laser beam and the nanoantenna array as the THG yield is predominantly originated from the field enhancement of the nanoantennas. Figure 4c illustrates the distribution of square unit cells containing nanoantennas in an array within a circular laser beam spot (dashed red line). Each nanoantenna is located at the center of its corresponding unit cell and the unit cell size is defined by the pitch distance. Nanoantennas which are located within ambiguous unit cells around the boundary of the beam spot (colored in gray) are more sensitive to beam position shift. Using this assumption, we further analyzed the effect of the ratio between the number of these ambiguous nanoantennas and the total number of nanoantennas within the focused beam diameter, which we found to be related to the instability of the THG yield enhancement.

To compute this ratio, we utilized the analytical die count estimation method, which is a well-known technique in the semiconductor industry for calculating the number of dies for a specific wafer size [27]. The analytical die count estimation is effective in counting the number of dies (square or rectangular shape) that can be made from a circular-shaped single wafer; the boundary pieces with non-square shapes cannot be used in a semiconductor process. Similarly, in our analysis, each nanoantenna can be assumed as a square unit cell (die) within a circular laser beam (wafer). We estimated that the increasing proportion of ambiguous unit cells of the total nanoantennas around the boundary of the beam spot (non-square shapes) increases the instability of THG. Here, the analytical die count estimation method is suitable for estimating the ratio between the number of ambiguous nanoantennas located at the beam boundary and the total number of nanoantennas within the beam diameter. The number of nanoantennas located at the boundary of the focused laser beam is given by

(4) $$N_{\text{boundary}}=\frac{2\pi \times r}{\sqrt{2\times d^{2}}}.$$

Using Eqs. (3) and (4), we define the ratio of ambiguity (*ROA*) as:

(5) $$\text{ROA}=\frac{N_{\text{boundary}}}{N_{\text{total}}}=\frac{\sqrt{2}\times d}{r}.$$

Equation (5) clearly shows that the pitch distance, *d*, of the nanoantenna array is closely correlated with the focused beam radius, *r*, in determining the *ROA*.

Figure 4d displays the total number of nanoantennas and the calculated *ROA* with radius = 3.15 and radius = 2.35 μm for varying pitch distances. The total number of nanoantennas decreases following the inverse square law according to Eq. (3) while the *ROA* increases linearly as the pitch distance increases for both radius values or focusing conditions. To further investigate the correlation between the *ROA* and the instability of the plasmonic THG yield enhancement, we calculated the coefficient of variation (CV) of the measured THG yield enhancement. The CV is a statistical measure of the spread of all measurements around the mean and represents the ratio between the standard deviation and the mean [28]. The THG enhancement instability under different focusing conditions cannot be directly compared because the experimental conditions, such as the input laser intensity and total number of excited nanoantennas, were different. On the other hand, the CV enables a quantitative comparison between different measurements regardless of the experimental conditions; therefore correlating the *ROA* with the CV is useful for investigating the instability of the THG yield enhancement. Figure 4e shows the correlation between the CV of *I*_sp_/*I*_bulk_ and the *ROA* with radius = 3.75, radius = 3.15 and radius = 2.35 μm. A larger focused beam radius of 3.75 μm was obtained using the same objective lens of radius = 3.15 μm (corresponding to NA = 0.25) because a smaller laser beam diameter was used at the entrance of the lens. Particularly, both the CV of *I*_sp_/*I*_bulk_ and *ROA* acquired from the three different focusing conditions coincide closely with each other. In addition, the CV of *I*_sp_/*I*_bulk_ increases roughly linearly with increasing *ROA* while both the CV of *I*_sp_/*I*_bulk_ and *ROA* tend to decrease with increasing focused beam radius for every pitch distance. The *ROA* is expressed by the ratio between pitch distance and beam radius, denoting the proportion of ambiguous unit cells (non-square shapes) of the total nanoantennas. The increasing *ROA* in Eq. (5) means the increase of ambiguous nanoantennas in the total number of nanoantennas. Thus, Figure 4e displays that the instability of THG is accelerated as the proportion of ambiguous nanoantenna increases. When the normalization by the active area is not considered for analyzing the THG power instability, the tendency of THG instability by the *ROA* increment is very similar. Figure S6 shows the power stability of plasmonic THG from the total third-harmonic power of nanoantenna array as a function of the *ROA* (see Supporting Information Section 8). Note that the conventional method for evaluating optical power stability was applied for displaying THG instability instead of the normalization by the area, still showing good agreement with our claim.

Thus, according to the above results, the stability of the plasmon-enhanced THG can be theoretically estimated in advance based on the *ROA*. For example, an *ROA* of less than 0.1 determines the maximum pitch distance of a nanoantenna array to be 100 nm if the beam radius is 1 μm, in order to yield a negligible CV for plasmon-enhanced THG experiments. Our results can support the deterministic design of a nanoantenna array to accomplish the requirements by considering scientific and non-scientific design factors. Conventional deterministic design means designing a system to satisfy the given requirements or risk-free systems [29, 30]. For example, when a steel sample can endure a certain force level for a steel tensile test, one can claim that this steel is deterministically designed to endure the applied force. In deterministic models, the model’s output is fully determined by the parameter and initial values to satisfy the requirement. Here, the requirement of a nanophotonic system based on the nanoantenna array could be a high photon yield, reliability, fabrication capability, or cost efficiency. The complete fulfillment of the aforementioned requirements is ideal, but it is practically not achievable. Our results provide a useful parameter for judging system reliability by revealing the maximum allowable pitch distance to satisfy the desired power stability. Then, the minimum pitch distance can be determined by considering other design requirements such as coupling effect, cost efficiency, or fabrication capability. For example, the unexpected coupling effect can occur when the pitch distance between nanoantennas is too short. Previous research reported that the plasmonic coupling effect decays over a length roughly 0.2 times the particle’s length [31, 32]. Further, dense nanoantenna fabrication requires state-of-the-art fabrication capability at a high cost. These design factors, including our technique, enable the deterministic design of pitch distance or optical configuration parameters (e.g., beam size) of the nanoantenna array to satisfy the tolerable stability of nanophotonic devices. Here, the *ROA* provides a useful and intuitive guideline for designing the nanoantenna arrays in terms of pitch distance and number of nanoantennas within a given focused beam size for maximizing plasmon-enhanced harmonic generation.

## 4 Conclusions

We have identified the shift of the focused laser beam position as the cause of the instability observed in the plasmon-enhanced THG yield from nanoantenna arrays and suggested a new method based on the *ROA* to minimize such power instability. We confirmed this by showing that the CV of the plasmonic THG yield enhancement is closely correlated with the *ROA* under three different focusing conditions.

The proposed method can be used to specify the pitch distance in a nanoantenna array before fabrication based on the *ROA,* facilitating a substantial simplification of the design process thus increasing the harmonic yield stability of nanoantenna arrays without requiring experimental tests. In most nonlinear experiments, the instability of the nonlinear signal can be caused by misalignment of the optical axis or uniformity of the fabricated nanoantennas. However, our experimental results show that the correlation between the pitch distance and beam size can also affect the third-harmonic power instability through a beam position shift. This method will be useful for exciting scientific applications of nonlinear nanophotonics based on arrayed nanoantennas, such as nanoantenna-enhanced nonlinear harmonic generation [9, 33], [34], [35], [36], [37], which is extremely sensitive to the field strength, two-dimensional array nonlinear metasurfaces [38–45] to steer light fields, and highly sensitive biosensors [46, 47] based on plasmonic field enhancement.

## Supplementary Material

Supplementary Material Details
