# Supplementary material for: Deterministic nanoantenna array design for stable plasmon-enhanced harmonic generation
Source: Nanophotonics. 2022 Oct 24;12(3):619–29. doi: 10.1515/nanoph-2022-0365 (PMC11501664; doi:10.1515/nanoph-2022-0365)
Supplement: Supplementary file 1 — Supplementary Material Details [file j_nanoph-2022-0365_suppl.pdf]

Supplementary Material

Deterministic nanoantenna array design for stable plasmon-enhanced harmonic generation

Tae-In Jeong[1,†], Dong Kyo Oh[2,†], San Kim[1], Jongkyoon Park[1], Yeseul Kim[2], Jungho Mun[2], Kyujung Kim[1,3], Soo Hoon Chew[1,3], Junsuk Rho[2,4,5,6]*, and Seungchul Kim[1,3]*

†Tae-In Jeong and Dong Kyo Oh contributed equally to this work.

[1] Department of Cogno-Mechatronics Engineering, College of Nanoscience and Nanotechnology, Pusan National University, Busan 46241, Republic of Korea

[2] Department of Mechanical Engineering, Pohang University of Science and Technology, Pohang, 37673, Republic of Korea

[3] Department of Optics and Mechatronics Engineering, College of Nanoscience and Nanotechnology, Pusan National University, Busan, 46241, Republic of Korea

[4] Department of Chemical Engineering, Pohang University of Science and Technology, Pohang, 37673, Republic of Korea

[5] POSCO-POSTECH-RIST Convergence Research Center for Flat Optics and Metaphotonics, Pohang, 37673, Republic of Korea

[6] National Institute of Nanomaterials Technology, Pohang, 37673, Republic of Korea

Corresponding Authors : Junsuk Rho (jsrho@postech.ac.kr) and Seungchul Kim (s.kim@pusan.ac.kr)

Section 1. Numerically calculated absorptance of an Au nanoantenna array

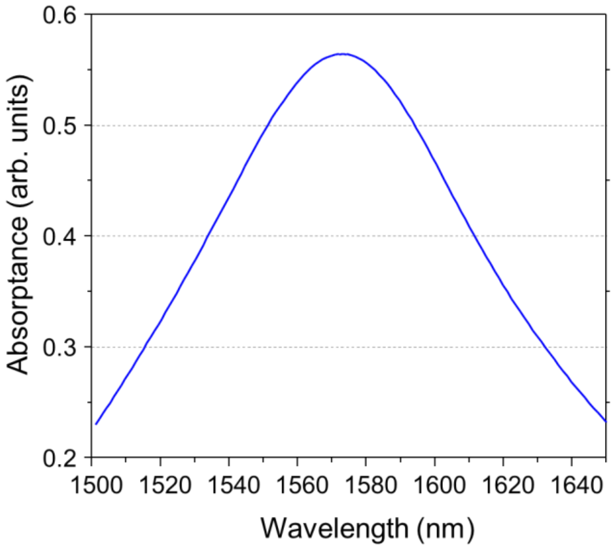

Figure s1. Finite-difference time-domain (FDTD) simulated absorption spectrum of an Au nanoantenna array on a Si substrate. Each nanoantenna has a length of 165 nm, width of 35 nm, and thickness of 40 nm with rounded corners.

Section 2. Fabrication process of Au nanoantenna arrays

The commercial Si on Al_2O_3 substrate used composed of a 500-nm-thick, (100) plane single-crystalline Si film grown on a single-crystalline R-plane Al_2O_3 substrate. The Au plasmonic nanoantenna arrays were fabricated parallel to the [110] orientation of the Si crystal on the substrate. An overview of the nanoantenna arrays patterned by e-beam lithography is given in Section 3. Firstly, a 250-nm-thick MMA positive-tone resist (MMA (8.5) MAA EL-8, MicroChem) was spin-coated (5000 rpm, 60 s) onto the substrate and heated at 150 °C for 5 min on a hotplate. Then, a 60-nm-thick PMMA positive-tone resist (950 PMMA A2, MicroChem) was spin-coated (2000 rpm, 60 s) onto the MMA layer and heated at 180 °C for 5 min on a hotplate, producing a MMA/PMMA bilayer structure. Using a standard EBL equipment (Elionix ELS-7800) with optimized writing parameters (acceleration voltage: 100 kV; beam current: 100 pA), the MMA/PMMA bilayer structure was exposed to an electron beam for nanoantenna patterning. In a subsequent solvent development process, the exposed areas were then subjected to different development rates in order to define clear deposition areas with a T-shaped profile. The MMA/PMMA bilayer was developed in a MIBK:IPA 1:3 solution at 4 °C for 18 min. After rinsing with IPA for 30 s and blowing with N_2 gas, the developed patterns were deposited first with Cr (3 nm) and later Au (40 nm) using electron beam evaporation (KVT, KVE-ENS4004), followed by a standard lift-off process.

Section 3. Scanning electron microscope images of Au nanoantenna arrays

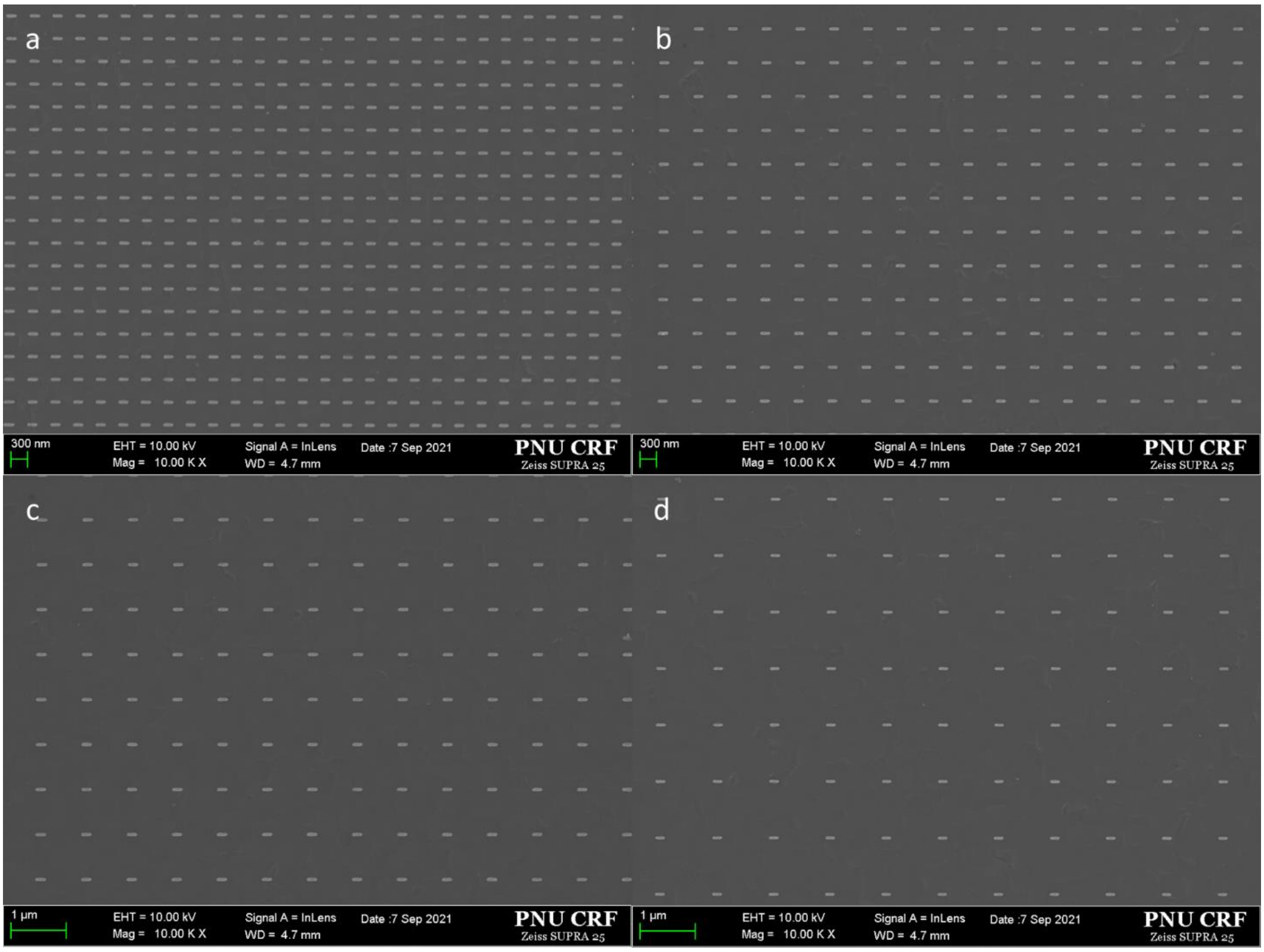

Figure s2. Scanning electron microscope images of nanoantenna arrays fabricated on a single-crystalline Si film grown on a Al_2O_3 substrate, with four different pitches of (a) 400 nm (b) 600 nm, (c) 800 nm, and (d) 1000 nm.

Section 4. Plasmonic field distribution at a depth of 5 nm inside the Si surface

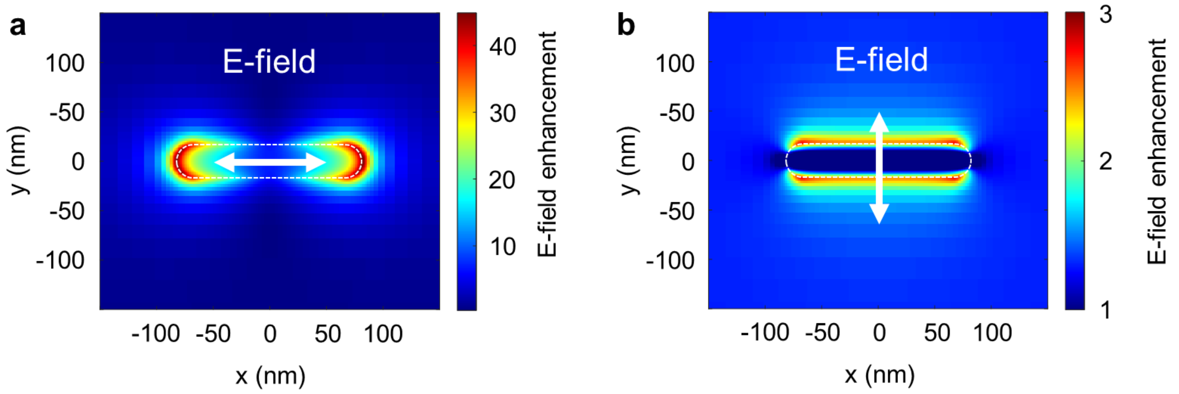

Figure s3. FDTD simulation results showing time-averaged plasmonic E-field enhancement for laser polarization (a) parallel and (b) perpendicular to the major axis of a nanoantenna, respectively. Simulations were calculated at a depth of 5 nm inside the Si surface. The white dashed line indicates the Au nanoantenna boundary.

Section 5. Estimate of the entire plasmon-enhanced area from a nanoantenna array

A_{active} denotes the plasmon-enhanced area of one nanoantenna while A_{inactive} denotes the bulk areas without plasmonic field enhancement in the nanoantenna array. These areas are given by

$$A_{\text{active}} = (S_{\text{length of rod}} + L_{\text{sp}}) \times (S_{\text{width of rod}} + L_{\text{sp}}) - (S_{\text{length of rod}} \times S_{\text{width of rod}})$$

$$A_{\text{inactive}} = (\pi \times r^2) - [N_{\text{total}} \times (S_{\text{length of rod}} + L_{\text{sp}}) \times (S_{\text{width of rod}} + L_{\text{sp}})],$$

where $S_{\text{width of rod}}$ and $S_{\text{length of rod}}$ are the width and length of a nanoantenna, respectively, L_{sp} is the length of the plasmon-enhanced area around each nanoantenna, and r is the focused beam radius. The entire plasmon-enhanced area within the focused beam area was calculated by multiplying the total number of nanoantennas, N_{total}, by the plasmon-enhanced area of each nanoantenna, A_{active}. L_{sp} was extracted from the FDTD simulation results.

Section 6. Power stability of the femtosecond laser

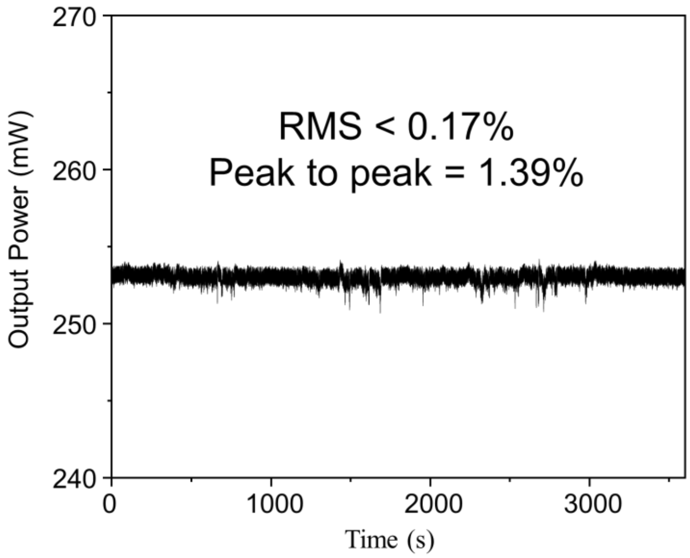

Figure s4. Power stability measurement of the femtosecond laser over one hour.

Section 7. Investigation of maximum and the minimum active areas

A_{active} × N_{total} \ Pitch distance	400 nm	600 nm	800 nm	1000 nm
Maximum active area	33489.3 nm^2	15006.71 nm^2	8523.45 nm^2	6347.25 nm^2
Minimum active area	32401.2 nm^2	14311.54 nm^2	7888.72 nm^2	4836 nm^2

Table 1. Maximum and minimum active areas at a focused beam radius of 2.35 μm (NA = 0.4)

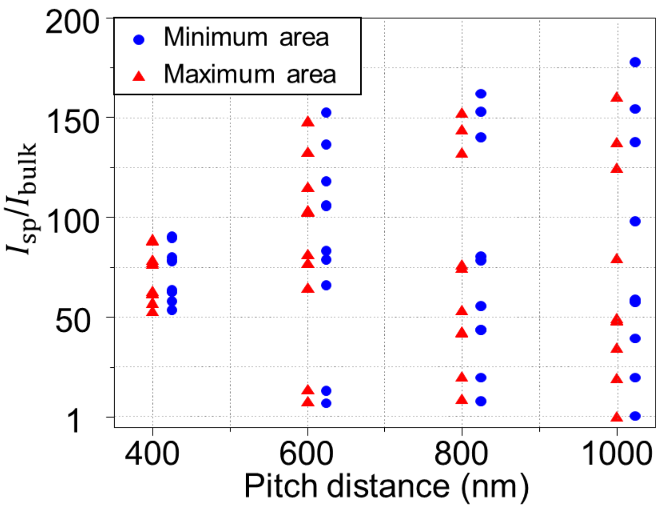

Figure s5. Calculated plasmonic THG yield enhancement for the maximum and minimum active areas. Minimum (blue circle) and maximum (red triangle) active areas were calculated for the focused beam radius of 2.35 μm (NA = 0.4).

Section 8. Optical power stability of the raw data

Figure s6. Correlation between the optical power stability of the raw data ($P_{sp} - P_{bulk}$) without the calculation process of equations (1)-(3) and *ROA*. We defined the optical power stability in percent, $(P_{max} - P_{min})/P_{avg} \times 100$. The P_{max} and P_{min} are the measured maximum and minimum third-harmonic power among the measurement data, respectively. The P_{avg} is the average of the measurement data for each experimental condition. The graph shows nearly the same tendency by the *ROA* increments, which is presented in Figure 4e.

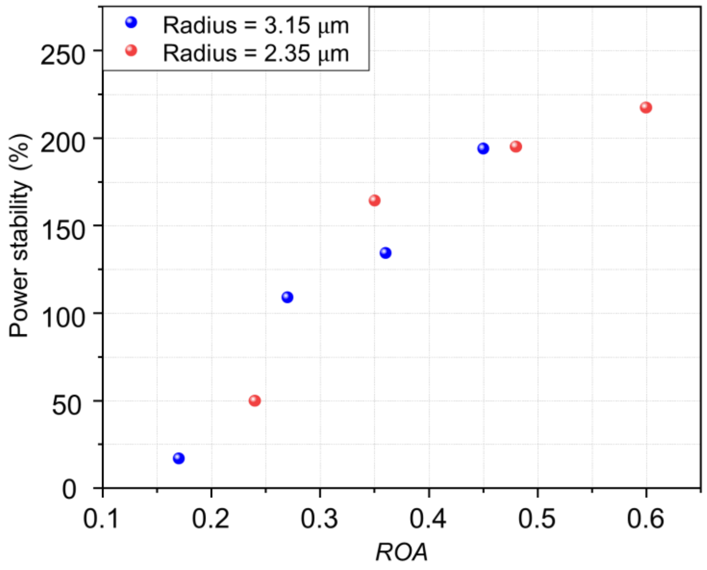

Figure s6. Optical power stability of ($P_{sp} - P_{bulk}$) as a function of the *ROA* with radius = 3.15 μm and radius = 2.35 μm.

Pitch distance / Test number	400 nm	600 nm	800 nm	1000 nm
1	13.8	3.75	4.48	4.03
2	15.7	1.94	5.4	1.83
3	15.2	6.6	2.28	5.98
4	14.8	5.08	0.7	5.3
5	15.1	6.35	4.07	4.01
6	14.5	5.34	4.27	0
7	14.54	7.31	5.84	4.21
8	16.39	5.13	5.86	1.22
9	15.39	3.76	3.17	3.63
10	16.34	3.98	2.31	0.63
P_{avg}	15.17	4.92	3.83	3.08
P_{max}	16.39	7.31	5.86	5.98
P_{min}	13.8	1.94	0.7	0
P_{max} - P_{min}	2.59	5.37	5.16	5.98
Power stability (%)	17.06	109.05	134.45	193.9

Table 2. (P_{sp} – P_{bulk}) data at a focused beam radius of 3.15 μm (NA = 0.25) Power unit in μV

Pitch distance / Test number	400 nm	600 nm	800 nm	1000 nm
1	30.9	23.07	12.72	3.11
2	26.98	18.1	3.74	0
3	22.06	21.13	13.76	1.13
4	26.91	2.15	6.72	9.1
5	21.95	10.08	3.73	7.11
6	19.95	16.08	11.73	2.11
7	19.2	13.2	1.8	3.15
8	30.9	12.1	4.74	5.11
9	22.14	1.17	6.78	3.14
10	28.1	16.07	0.77	8.13
P_{avg}	24.9	13.31	6.64	4.2
P_{max}	30.9	23.07	13.76	9.1
P_{min}	19.2	1.17	0.77	0
P_{max} - P_{min}	11.7	21.9	12.98	9.1
Power stability (%)	46.97	164.47	195.27	216.66

Table 3. (P_{sp} – P_{bulk}) data at a focused beam radius of 2.35 μm (NA = 0.4) Power unit in μV
